# Comprehensive pain management as a frontline treatment to address the opioid crisis

**DOI:** 10.1002/brb3.2369

**Published:** 2021-09-23

**Authors:** Joseph Gregory Hobelmann, Andrew S. Huhn

**Affiliations:** ^1^ Department of Psychiatry and Behavioral Sciences Johns Hopkins University School of Medicine Baltimore Maryland USA; ^2^ Ashley Addiction Treatment Havre de Grace Maryland USA

**Keywords:** addiction, pain, psychiatry, psychology

## Abstract

Background: The opioid crisis continues to devastate individuals and communities in the United States and abroad. While there have been several measures to address the over‐prescription of opioid analgesics, the number of overdose deaths related to prescription opioids has not changed appreciably in the last 10 years. Comprehensive (or multidisciplinary) pain recovery programs consist of providers from multiple backgrounds that treat pain on an individual level through a combination of approaches including physical therapy, emotional and spiritual support, cognitive behavioral therapy, and non‐opioid pharmacotherapies. Because there is a dynamic interplay between a given chronic pain patient and multiple providers, comprehensive pain programs are not as “standardized” as other medical treatments because they are meant to meet the individual needs of each patient and their specific pain diagnoses

Methods: Review of the literature.

Results: There is evidence that comprehensive pain treatment can reduce pain severity and improve functioning; comprehensive pain treatment can be used to treat those with post‐surgical pain, thus preventing the onset of non‐medical opioid use and opioid use disorder, and in persons with chronic pain who are on long‐term opioid therapy, as a method to reduce or eliminate opioid medication use. Comprehensive pain recovery programs were abundant for a period from the 1960s through the 1980s, but for a variety of reasons, they became financially unsustainable as the national reimbursement environment evolved.

Conclusions: In the context of the protracted and deadly opioid crisis, revitalizing and expanding comprehensive pain treatment should be considered as a frontline approach to treat chronic pain.

## Abstract 2

Prescription opioids play a major role in the opioid crisis, yet long‐term opioid therapy is often ineffective in treating chronic pain. Comprehensive pain treatment is a multidisciplinary approach to pain treatment that favors combinations of psychological and physical therapies to treat pain without opioid analgesics. Increasing access to comprehensive pain treatment is a logical step in reducing the availability of prescription opioids and addressing the deadly and protracted opioid crisis in the United States.

## 1 INTRODUCTION

Prescription opioids continue to play a major role in the protracted opioid crisis in the United States. The majority of persons with opioid use disorder begin their non‐medical opioid use with prescription opioids, and there is a pressing need to employ macro‐level changes to the medical treatment of chronic pain that reduce the reliance on opioid analgesics (Vadivelu et al., 2018). Prescription opioid overdose deaths increased approximately fourfold between 2000 and 2010, and have not changed appreciably since that time despite coordinated public health efforts to curb opioid prescription (National Institute on Drug Abuse, 2021). Overall, the United States has experienced a moderate reduction in the total number of opioid prescriptions and in the number of adults receiving an opioid prescription in recent years. However, there has also been an increase in the mean duration of opioid prescriptions and the number of prescriptions for 30 days or longer (Schieber, 2020). Guidelines to mitigate the risk of addiction and overdose in prescribing opioids for chronic pain include limiting daily doses to 90–200 mg of morphine equivalents, using caution when titrating an opioid dose, and reducing doses by 25–50% when switching between different opioid medications (Nuckols & Chou, 2014). The Centers for Disease Control and Prevention (CDC) also note that non‐opioid therapies are now preferred for the treatment of chronic pain (Dowell et al., 2016). However, there is no one‐size‐fits‐all approach among non‐opioid strategies for the treatment of chronic pain.

Chronic pain may stem from an acute pain episode (e.g., an injury causing lasting musculoskeletal pain) or disease state (e.g., diabetic neuropathy), yet there is often no identifiable etiology in chronic pain syndromes. As such, chronic pain treatment is challenging as there is significant variability regarding the source of pain. As chronic pain persists, it affects people not only physically, but also emotionally, cognitively, and spiritually. There is evidence that comprehensive pain treatment can reduce pain severity and improve functioning, following opioid cessation (Townsend et al., 2008), and reduce opioid use following surgery (Thuener et al., 2020; Townsend et al., 2008). Many of the components of comprehensive pain treatment have shown independent efficacy, yet comprehensive pain treatment is, by definition, a dynamic process with the goal of finding the optimal combination of therapies to treat pain at an individual level. Below, we outline the evidence supporting independent components of comprehensive pain treatment, as well as future directions aimed at utilizing comprehensive pain treatment more broadly as a frontline approach to reduce or eliminate the need for long‐term opioid therapy in the majority of chronic pain patients.

## 2 HISTORY OF MULTIDISCIPLINARY PAIN TREATMENT

Practitioners who treat pain emanate from a variety of disciplines (medicine, anesthesiology, psychiatry, psychology, physiatry, surgery, nursing) and may feel underqualified to be solely responsible for treating the complex nature of chronic pain (Nicolson et al., 2009). Comprehensive (or multidisciplinary) pain recovery programs typically consist of providers from multiple backgrounds including pain medicine, internists, psychologists, psychiatrists, physical therapists, occupational therapists, addiction counselors/specialists, vocational rehabilitation specialists, and others, working together to create a common treatment plan for each patient (Chien et al., 2019). Because there is a dynamic interplay between a given chronic pain patient and multiple providers, comprehensive pain programs are not as “standardized” as other medical treatments because they are meant to meet the individual needs of each patient and their specific pain diagnoses. These programs were abundant for a period from the 1960s through the 1980s and were shown to improve primary pain and disability outcomes (Kamper et al., 2014). For a variety of reasons, they became financially unsustainable as the national reimbursement environment evolved. Treatment for patients with chronic pain was subsequently focused on the physical aspects of pain, which included procedural interventions and medical management, generally with analgesic medications such as opioids (Tompkins et al., 2017). In addition, reimbursement for “cognitive/emotional specialties” was limited to such an extent that patients suffering from chronic pain rarely received psychological intervention for treatment of self‐reported chronic pain. Finally, “alternative therapies”, such as novel medications, acupuncture, massage, and meditation, have been underutilized due to insufficient reimbursement, but often have as much proven efficacy as many of the medically accepted treatments, such as epidural steroid injections (Hilton et al., 2017; Stoll & Sanchez, 2002; Tang et al., 2018; Vickers & Linde, 2014).

As the number of comprehensive pain programs dissipated, there grew a larger reliance on medications and surgical procedures as the primary modes of treatment. This was in large part due to mandates requiring universal assessment and treatment of pain (Tompkins et al., 2017). Although these mandates were created with good intentions, they resulted in an overreliance on long‐term opioid therapy for the treatment of chronic pain. Opioids were not only prescribed by well‐meaning providers to treat pain, but also by a variety of providers looking to make a profit. As a result, the access to prescribed opioids increased dramatically and with that came an increase in persons with opioid use disorder. This created a larger demand for opioids, which increased the use of illicit opioids and ultimately, created the current opioid crisis (Vadivelu et al., 2018). While it is clear that opioids are effective in the treatment of acute pain, there have been several prospective studies indicating poor long‐term analgesic and health effects of chronic opioid therapy (Ballantyne, 2017). As such, it is clear that alternatives should be sought and it is time to revisit the concept of comprehensive pain treatment in earnest.

## 3 PSYCHOLOGICAL FACTORS IN CHRONIC PAIN

Chronic pain becomes particularly debilitating when a person's primary identity has become that of a “pain patient.” The best predictor of acute pain transitioning to chronic pain is a negative emotional response to the pain (Casey et al., 2008). As pain persists, function can deteriorate, leading to a lower perceived quality of life. With this comes a diminished capacity to tolerate pain, which in turn continues to worsen daily functioning and quality of life. Over time, the pain becomes the primary focus of life and the assumed identity changes to that of a person suffering with chronic pain.

In the context of identifying as a “pain patient,” both primary and secondary gains are often present. Primary gain refers to the internal factors that maintain pain (Eisendrath, 1995). For example, having pain reduces the emotional strain of not being able to function at normal capacity. Secondary gain refers to external factors that are often easier to identify. For example, continued pain excuses individuals from previous responsibilities, such as work or duties at home (Cole, 1993). Furthermore, individuals suffering from debilitating chronic pain tend to lose their sense of self‐efficacy, and an individual's locus of control may transform from internal to external. By focusing on an external locus of control, individuals might feel that everything is done to them rather than having a sense that outcomes in life are directly related to their thoughts and behaviors. Caring for the patient can become the primary focus of the family, making chronic pain a true family illness. Thus, an individual with chronic pain may experience a narrowing of their repertoire of daily activities as they minimize social activities and no longer participate in previously enjoyable endeavors. This can lead to a feeling of isolation and potential emergence and/or worsening of psychiatric comorbidities. The development of the role of a “pain patient” is insidious and often goes unrecognized by both the person and caregivers (Miaskowski et al., 1997). Patients are not actively seeking this role and should not be blamed for its evolution. True progress can be made when patients realize that external forces cannot simply fix them. They must realize that what they have been doing is not working and a fundamental change in thinking and behavior is needed to improve their situation. Taking ownership of their pain and playing an active role in their recovery can lead to remarkable results.

In addition, there is a relationship between the physical experience of chronic pain and negative emotional states. However, the direction of this relationship is complex and difficult to fully characterize. For example, instances where pain leads to depressive and/or anxiety symptoms or vice versa (Eisendrath, 1995). There is a bidirectional relationship between unpleasant affective states and decreased pain thresholds (Leknes & Tracey, 2008; Stanos & Houle, 2006), and the neuroanatomical indicators of pain and depression have a considerable overlap. For example, brain structures involved in both pain and depressive symptoms include the insular cortex, prefrontal cortex, hippocampus, and amygdala, and decreased cortical volume and synaptic function are evident in both chronic pain individuals and in persons with major depressive disorder (Goesling et al., 2013). Pain and depression also share common neurochemical pathways as both serotonin and norepinephrine are involved in depression, anxiety, and in the modulation of pain signals (Bair et al., 2003). Anxiety and pain are also linked to several other physiological phenomena, such as muscle tension and tachycardia that can worsen both conditions. Finally, treating depressive and anxiety symptoms in patients with somatic complaints reduces the intensity of pain (Lenze et al., 2005).

## 4 MODERN APPROACHES TO COMPREHENSIVE PAIN TREATMENT

Comprehensive pain treatment begins with a thorough evaluation of the individual, their pain diagnoses, and current and past psychiatric conditions (Chien et al., 2019). After assessing for chronic pain and comorbid psychiatric issues, a treatment plan is created, considering the physical, emotional, cognitive, and spiritual difficulties. Finally, a continuing care plan is developed, taking into account the cognitive and behavioral changes needed to sustain lasting improvement. This compressive approach should include physicians and medical professionals, physical therapists, mental health professionals and social workers, and spiritual counselors, as appropriate. Treatment should be tailored to the individual, with a primary care provider (or other identified provider) coordinating the intensity and duration of various treatments as it provides organization and consistency.

All efforts should be made to resolve any physical defects causing the pain, if they can be identified. A full interventional workup should be completed to evaluate for surgeries, injections, or other procedures that may help attenuate the pain. Several types of injections, such as epidural steroid injections, nerve blocks, or facet injections, can be performed for diagnostic as well as therapeutic purposes. Surgical procedures, such a discectomy and spinal fusion are meant to be more definitive, but are not always successful. Spinal cord stimulators are reserved for refractory cases of radiculopathy and complex regional pain syndrome (CRPS) and have demonstrated moderate effect (Song et al., 2014).

There are several “alternative treatments” that are frequently utilized in comprehensive treatment programs. These are therapies that are not typically taught in medical school because they have not been thoroughly evaluated by the standards of Western medicine, and as such are often not reimbursed by insurance companies. However, anecdotally, they can be very effective in well‐selected individuals. These therapies include acupuncture, massage, music, and transcutaneous electrical nerve stimulation (TENS). Recently, there has been interest in the analgesic effect of repetitive transcranial magnetic stimulation (rTMS); however, more evidence is needed to determine the time and place for its clinical application as an analgesic (Galhardoni et al., 2015).

Physical therapy is an essential part of recovery from musculoskeletal chronic pain. For many individuals with chronic musculoskeletal pain, there is a reduction in daily physical activity that results in them becoming deconditioned. The process of deconditioning and reduction in daily activity can, in turn, worsen pain. As such, resuming normal activity should be an immediate goal (conditioning), and delaying the resumption of normal activity may lead to greater disability. Treatment with physical therapy aims to reduce disability and suffering by reducing pain and increasing tolerance to movement. Physical therapy, aqua therapy, and exercise to improve range of motion and strength can improve function, pain, and quality of life outcomes in patients with chronic pain; this may lead to an overall improvement in mood, including a reduction in depression and anxiety symptoms (Allen, 2006; Geneen et al., 2017). Indeed, the importance of physical therapy and exercise must be stressed as an essential component of a comprehensive pain recovery program. Pain, depression, and anxiety are mutually maintaining, and treatment of both pain and comorbid psychiatric symptoms is necessary to achieve optimal outcomes (Goesling et al., 2013).

Many medications used primarily to treat psychiatric conditions also alleviate pain. These include antidepressants primarily, but in some cases mood stabilizers (anticonvulsants) may also be of benefit (McQuay & Moore, 1997). For patients with co‐occurring pain and depression or anxiety, treatment with antidepressants that increase serotonin and norepinephrine in the synaptic cleft can be beneficial to pain outcomes. Tricyclic antidepressants (TCAs), such as amitriptyline, nortriptyline, and desipramine, have a well‐established efficacy in treating neuropathic pain (Dosenovic et al., 2017). Serotonin and norepinephrine reuptake inhibitors (SNRIs), like TCAs, increase the availability of monoamines in the synaptic cleft, and have been shown to be effective in a variety of pain conditions and have the advantage of better side‐effect profiles when compared with TCAs (Dosenovic et al., 2017). Mood stabilizers work to reduce pain through varied mechanisms, but they all essentially decrease excitation in neurons, which is thought to mediate their analgesic effects. In addition, they are effective for the treatment of bipolar affective disorder in chronic pain patients. The commonly used mood stabilizers that also have demonstrated analgesic effect include valproic acid, carbamazepine, lithium, gabapentin, and pregabalin (Dosenovic et al., 2017).

Antidepressant medication can effectively treat depression in the presence of chronic pain, yet there is evidence from a large clinical trial on collaborative care interventions that pain has a negative impact on treatment response for persons with major depressive disorder or alexithymia, and that resolution of pain is strongly correlated with resolution of depressive symptoms (Kroenke et al., 2008). When mood disorders accompany chronic pain, as when they accompany other chronic medical disorders, there may be some extra hurdles in improving patient outcomes. These include aversive physical symptoms, severe deactivation, vocational dysfunction, marital conflict, social isolation, and concurrent medications. Comprehensive assessment of these issues and the formulation of a treatment plan that takes them into account increases the likelihood of successful depression treatment in chronic pain patients. Pain often subsides with improvement in depressive symptoms, and patients will typically report that they may still have pain, but that they can better tolerate pain and function in their daily lives.

Opioids deserve special attention as controversy surrounds long‐term treatment due to concerns regarding long‐term efficacy and safety, including the development of tolerance, dependence, abuse, overdose, and opioid‐induced hyperalgesia (Harned & Sloan, 2016). In individuals with psychiatric comorbidity, opioid use must be considered even more carefully, as opioids may exacerbate depressive and anxiety disorders (Scherrer et al., 2016). Pain typically improves when opioids are discontinued in patients who have been using them for long‐term treatment (Crisostomo et al., 2008). Hence, discontinuation of opioids is an important initial step in a comprehensive treatment program.

In addition to medical and interventional modalities, psychotherapeutic approaches are essential for optimal treatment of the emotional aspects of chronic pain. The clinical art of emotional treatment for those with chronic pain consists of establishing a solid therapeutic alliance around those problems. There is a larger body of evidence to support the use of cognitive behavioral therapy (CBT) for both emotional disturbances as well as chronic pain (Knoerl et al., 2016). Since the initial applications of CBT to chronic pain, much research has established the importance of cognitive and behavioral processes in how individuals adapt to chronic pain. Over the last three decades, CBT has become a first‐line treatment for individuals with chronic pain. There is ample evidence of its efficacy in improving pain and pain‐related problems across a wide spectrum of chronic pain syndromes. The benefits of CBT have been found to continue up to six months after the completion of active treatment sessions (Knoerl et al., 2016). In order to be effective across practitioners and sites, CBT should be delivered in a structured fashion by a trained provider in individual and/or group settings.

Spirituality is difficult to define as it has different meanings to individuals depending on multiple factors, including culture, background, and religion. However, it can be described in general as the core of a person's understanding of a higher power, and/or identity and motivation for life. As described above, chronic pain slowly and insidiously transforms a person's identity until they are left only with the identity of a person in pain (Rippentrop et al., 2005). This can result in drastic changes in motivation for living. A person who was once motivated to have a family, build a career, and develop meaningful relationships can be reduced to being motivated solely to cope with their pain and distress (Rippentrop et al., 2005). In this state, they tend to lose connection to others and are left feeling isolated, whether or not they have a strong support system. Their ability to cope and tolerate distress is diminished. Spiritual therapy can be defined as any modality that will help return meaning and purpose to one's life and reshape their identity. Spiritual care may provide insight, inspire hope, and is thought to mediate mental and physical health for patients (Sorajjakool et al., 2006). There is a paucity of empirical research in this area, but it makes intuitive sense that this is beneficial in patients with pain, with or without comorbid psychiatric illness (Garschagen et al., 2015).

Meditation as a therapeutic option bridges emotional, cognitive, and spiritual domains of comprehensive pain treatment, and has been shown in several studies to relieve the perception of pain (Hilton et al., 2017). Meditation can be broadly defined as a form of mental training that aims to improve an individual's core psychological capabilities, such as attention and emotion regulation. In particular, mindfulness meditation has received much recent attention as a potential therapeutic modality. There is mounting evidence of the therapeutic effects of mindfulness on pain, stress, negative emotion, and addictive processes (Hilton et al., 2017). Meditation can help focus patients on the present and provide inspiration for their immediate recovery needs.

## 5 MOVING THE FIELD OF PAIN TREATMENT FORWARD

Improving pain treatment in the United States requires a nuanced approach in patient management and a reduced reliance on opioid analgesics. Comprehensive pain treatment is a dynamic process that has been shown to be effective in improving pain, mood, and function in patients with chronic pain. By definition, comprehensive pain treatment offers core medical and psychosocial support, along with a buffet of alternative treatment options to meet the needs of patients on an individual level. This approach has consistently been shown to be associated with return to work, reduction in hospitalization over a 10‐year period, and meaningful, sustained improvement in pain and function (Patrick et al., 2004). In addition, there is evidence that comprehensive pain treatment is not only efficacious, but also cost‐effective in the long‐term (Gatchel & Okifuji, 2006). One of the focus areas of the National Institutes of Health's (NIH) Helping to End Addiction Long‐term (HEAL) initiative is clinical research in pain management. Within this initiative, the evaluation of innovative therapies for pain management, their combinations, and health service‐level assessments of the value of comprehensive pain treatment, has the potential to directly address the current opioid crisis, reserving long‐term opioid therapy for those who truly need it, but funneling the majority of persons with chronic pain into alternative therapies that improve pain directly and support long‐term health.

## CONFLICT OF INTEREST

Andrew S. Huhn receives research funding from Ashley Addiction Treatment through his university. Joseph Gregory Hobelmann reports no conflict of interest.

## AUTHOR CONTRIBUTIONS

Joseph Gregory Hobelmann conceptualized the manuscript and Joseph Gregory Hobelmann and Andrew S. Huhn drafted, refined, and edited the manuscript.

### PEER REVIEW

The peer review history for this article is available at https://publons.com/publon/10.1002/brb3.2369.
